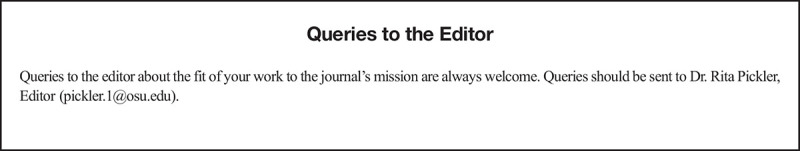# Nurse Leaders’ Interpersonal Communication Competence

**DOI:** 10.1097/NNR.0000000000000837

**Published:** 2025-05-19

**Authors:** Petra Kämäräinen, Leena Mikkola, Anu Nurmeksela, Tarja Kvist

**Affiliations:** **Petra Kämäräinen, MHSc, RN** is Doctoral Researcher, Department of Nursing Science, University of Eastern Finland, Kuopio, Finland.; **Leena Mikkola, PhD** is Professor, Department of Communication Sciences, Tampere University, Tampere, Finland.; **Anu Nurmeksela, PhD, RN** is University Lecturer, Department of Nursing Science, University of Eastern Finland, Kuopio, Finland.; **Tarja Kvist, PhD, RN** is Professor, Department of Nursing Science, University of Eastern Finland, Kuopio, Finland.

**Keywords:** communication, health services administration, leadership, nursing leadership, qualitative research

## Abstract

**Background:**

Despite the beneficial associations of nurse leaders’ interpersonal communication competence with job satisfaction, engagement, and reduced burnout among nurses, there has been little research on the topic.

**Objectives:**

To describe nurse leaders’ perceptions of interpersonal communication competence.

**Methods:**

A qualitative descriptive study was conducted in the three well-being services counties in Finland between February and April 2024. A total sample of 21 nurse leaders participated. Data were analyzed using abductive content analysis, starting with a deductive phase guided by a conceptual framework of nurse leaders’ interpersonal communication competence categories, followed by inductive analysis. Furthermore, the outcome of the analysis was viewed through the theory of interpersonal communication competence.

**Results:**

In addition to the three categories of nurse leaders’ interpersonal communication competence—message competence, relational competence, and task competence—the study identified a fourth main category: ethical principles. Alongside these main categories, 27 subcategories were identified. An exploration of the results through the lens of interpersonal communication competence theory showed that the theory’s cognitive, behavioral, and affective dimensions were identifiable in the description.

**Discussion:**

Using a unique multidisciplinary methodological solution, the results highlight the complex and adaptive nature of nurse leaders’ interpersonal communication competence. It provides a thorough description of nurse leaders’ interpersonal communication competence, supported by interpersonal communication competence theory. The results promote an understanding of the interpersonal communication competence required in nursing leadership. To retain competent nurses and ensure quality care, a comprehensive understanding of communication competence in nursing leadership is essential, highlighting the need for further research on this topic.

Effective leadership communication in the nursing environment is pivotal for nurse retention and preventing burnout (Jankelová & Joniaková, 2021; Stillman et al., 2024; Tang & Hudson, 2019). Leaders’ transparent and regular communication, empathy and respect, and effective teamwork reduced burnout among healthcare professionals during the COVID-19 pandemic (Stillman et al., 2024). Recent studies have shown that nurse leaders’ communication skills significantly affect nurses’ job satisfaction and engagement (Fowler et al., 2021; Jankelová & Joniaková, 2021), reduce turnover (Lee & Kim, 2020), and extend nursing careers (Nurmeksela et al., 2023).

Despite multiple beneficial connections, little research exists on nurse leaders’ interpersonal communication competence (Kämäräinen, Mikkola, Nurmeksela, Wright, & Kvist, (2024)). A systematic review identified three categories of nurse leaders’ interpersonal communication competence: message competence, relationship competence, and task competence (Kämäräinen, Mikkola, Nurmeksela, Wright, & Kvist, (2024)). Message competence refers to the core communication skills essential for nursing leadership. Whereas relational competence involves establishing, developing, and maintaining effective professional relationships; task competence focuses on interpersonal competence to achieve tasks and goals in nursing leadership. The review found that research has concentrated on describing communication skills rather than competence, limiting the understanding to the behavioral level (Kämäräinen, Mikkola, Nurmeksela, Wright, & Kvist, (2024)).

To better understand nurse leaders’ interpersonal communication competence, this study explored it as described by the nurse leaders themselves. Using categories from the previous review (Kämäräinen, Mikkola, Nurmeksela, Wright, & Kvist, (2024)) and examining them through the lens of the interpersonal communication theory (Spitzberg, 2022), described below, the objective was to produce new information to understand the construct of nurse leaders’ interpersonal communication competence to help identify, assess, and develop the competencies required in nursing leadership.

Although communication skills and competence are often used synonymously, competence is generally considered broader than skills (Hannawa & Spitzberg, 2015). It consists of three inextricably linked dimensions: cognitive, behavioral, and affective (Spitzberg, 2022). When considering communication competence within interpersonal interaction and relationships, it can be examined through the concepts of interpersonal or relational competence (Kokkonen & Koponen, 2020). This research focuses on examining the topic through interpersonal communication competence, which refers to an individual’s ability to accomplish personal objectives through interpersonal communication, whereas relational competence relates to the ability to maintain a relationship with a partner (Hannawa & Spitzberg, 2015).

Considering the dimensions of interpersonal competence, competent communication requires mastery of all three. A communicator cannot be considered competent if they lack knowledge about the content and process dynamics of communication (cognitive), skills for message production or interaction (behavioral), or motivation to communicate (affective; Spitzberg, 2022). The theory also identifies a metacognitive dimension, which includes metacognitive know-how (Hannawa & Spitzberg, 2015).

Interpersonal communication competence is commonly defined as the degree to which meaningful behavior is considered appropriate and effective in achieving intended outcomes. While effectiveness assesses how well communication accomplishes desired results, appropriateness assesses how well communication fits into the situation and context (Horila, 2020; Spitzberg, 2022). Ethical principles state that communication should not threaten or offend any parties involved (Kokkonen & Koponen, 2020) and can be considered within these criteria: If communication is effective and appropriate, it is likely to be ethical (Spitzberg & Cupach, 2011).

Communication appropriateness and effectiveness differ among professions, as do perceptions of competent communicators (Dannels, 2001; Horila, 2020). For example, immediacy may not be considered the priority in engineering, but it is crucial to a nurse’s communication competence (Dannels, 2001). Researchers’ interest in interpersonal communication competence in work settings has increased in recent decades. The research has focused on profession-specific interpersonal competencies, such as nurses (Noh & Kang, 2024), university teachers (Huerta-Soto et al., 2024), and entrepreneurs (Cegarra-Navarro et al., 2024). Nevertheless, extending research into other professional contexts, including nursing leadership, it is important to explore the meanings and expressions of communication and improve our understanding of interpersonal communication competence (Kokkonen & Koponen, 2020).

## METHODS

### Aim and Research Question

The study aimed to describe nurse leaders’ perceptions of interpersonal communication competence. The research question was: “How do nurse leaders describe interpersonal communication competence in nursing leadership?”

### Design

The study was an interview study (Kyngäs et al., 2020). Reporting follows the Consolidated Criteria for Reporting Qualitative Studies checklist (Tong et al., 2007), and the study protocol was registered in the Open Science Framework Register on February 18, 2024 (Kämäräinen, Mikkola, Nurmeksela, Wright, & Kvist, (2024)).

### Study Setting and Data Collection

Purposive sampling was used to recruit nurse leaders from three well-being services counties in Finland. In the Finnish tax-funded public health system, 21 well-being services counties organize health services for the entire population, along with social welfare and rescue services. Given the variations in size, service structure, and organization of nursing leadership across the counties, three different sizes and locations of counties with clear nursing leadership structures were selected for the study. In the selected counties, nurse leaders manage nursing work in social and healthcare services. Inclusion criteria were nurse managers, nurse directors, or nurse leaders with a similar title in the healthcare sector, recognized for good interpersonal communication competence. The chief executive nursing officers of the well-being services counties facilitated the recruitment process by forwarding the invitation to the nurse leaders they considered suitable for the study. Prior to recruitment, they were informed about the study’s aim to describe nurse leaders’ interpersonal communication competence categories—message competence, relational competence, and task competence (Kämäräinen, Mikkola, Nurmeksela, & Kvist, 2024)—through the cognitive, behavioral, and affective dimensions of interpersonal communication competence (Spitzberg, 2022). Considering the study group, they were familiarized with the subjective nature of assessing interpersonal communication competence. After receiving the invitation, interested nurse leaders contacted the first author. All 21 contacted nurse leaders were interviewed between February and April 2024. Data saturation was achieved by the 19th interview, with the last two interviews complementing earlier data.

The first two interviews were used to pilot test the interview guide (Supplemental Digital Content [http://links.lww.com/NRES/A567], File 1). As the pilot interviews were consistent with the subsequent interviews, they were included in the data analysis. All interviews followed the interview guide that included themes and open-ended questions about the nurse leaders’ interpersonal communication competence. Interview themes were guided by the previous systematic review results (Kämäräinen, Mikkola, Nurmeksela, Wright, & Kvist, (2024)), along with the interpersonal communication competence theory (Spitzberg, 2022).

Interviews lasted between 47 and 94 min, averaging 77 min. The first author conducted interviews face-to-face or remotely, based on the nurse leader’s preference. They were recorded with permission using audio (face-to-face) and video (remote), stored securely, and transcribed using Microsoft’s transcription tool. Transcribed interviews were verified against the recordings. A total of 394 pages of raw data were produced using Calibri size 11 font and single line spacing. All personal details were removed from the reports.

### Data Analysis

**FIGURE 1:**
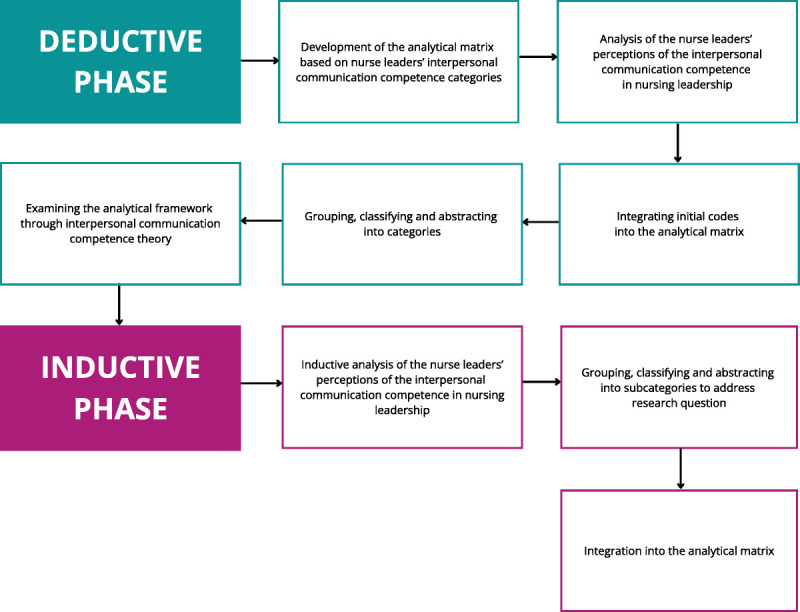
The analysis process. *Note*. The figure illustrates the abductive content analysis process. It begins with a deductive analysis using categories from Kämäräinen, Mikkola, Nurmeksela, and Kvist’s (2024) systematic review. The data were analyzed inductively within these main categories, coded, and classified into subcategories. The deductive phase concluded with an examination of the results using interpersonal communication competence dimensions (Spitzberg, 2022). Finally, the inductive phase entailed coding, grouping, and abstracting the data. The resulting categories were integrated into the analytical matrix.

The data were analyzed using abductive content analysis to achieve a comprehensive and in-depth understanding (Graneheim et al., 2017). The starting point was a deductive content analysis using the competence categories from Kämäräinen, Mikkola, Nurmeksela, Wright, & Kvist, (2024) systematic review as an analytical matrix (Figure 1). Data were then carefully examined to create an accurate overview. Within the deductively defined main categories, the data were analyzed inductively. The analysis focused on spoken content only. The unit of analysis was a phrase, a sentence, or a multisentence idea. After coding, initial codes were transferred to the matrix and classified into subcategories. Finally, the subcategories that emerged from the analysis were examined using the dimensions identified by the interpersonal communication competence theory (Spitzberg, 2022).

A closer examination of the data revealed that in addition to the cognitive, behavioral, and affective dimensions, an ethical dimension emerged from the data, which has also been identified in the literature (Spitzberg & Cupach, 2011). The ethical dimension was analyzed inductively, first by coding, then by grouping and abstracting (Graneheim et al., 2017). As the ethical dimension described the ethical principles relevant to nurse leaders’ interpersonal communication competence, it was descriptively named “ethical principles” (Figure 1).

The first author performed the analysis using Atlas.ti software. The initial analysis was conducted in Finnish, as all interviews were conducted in Finnish. The research team discussed and refined the codes and their English translations to ensure alignment with communication literature. To maintain meanings of quotations, the English translations were translated back into Finnish and compared with the originals. During data abstraction and categorization, 3,209 open codes, 94 themes, and 27 subcategories were created.

### Trustworthiness

Six criteria were used to ensure this study’s trustworthiness: credibility, transferability, dependability, confirmability, authenticity (Connelly, 2016), and reflexivity (Korstjens & Moser, 2018). The research team’s familiarity with the topic enhanced credibility and confirmability (Connelly, 2016; Korstjens & Moser, 2018). Considering credibility, member checking was not employed. Triangulation was improved by combining deductive and inductive analytical methods (Korstjens & Moser, 2018). Original expressions added the transparency of the analysis process (Kyngäs et al., 2020). The research strived for dependability with a clear, well-documented process and enhanced transferability by presenting the analysis and results truthfully and in detail (Connelly, 2016). Considering reflexivity, the participants were seen as valuable informants on the topic. The multidisciplinary research team—composed of a female doctoral researcher (principal investigator) and three female PhDs (co-investigators/supervisors)—brought diverse backgrounds in communication and nursing science (Korstjens & Moser, 2018).

### Ethical Considerations

All participating counties with well-being services were given permission to conduct the research. The study followed the guidelines of the Finnish National Board on Research Integrity (2019) and the EU General Data Protection Regulation (EUR-Lex, 2016). According to the ethical regulations governing Finnish research, ethical approval is not required for interview studies that do not involve patients, do not cause harm, and do not intervene in bodily integrity.

The study invitation informed participants about the study’s purpose, data management, confidentiality, anonymization, and their right to withdraw consent at any time. Participants received study information after giving verbal consent to participate. To ensure confidentiality and avoid identifying nurse leaders, the nurse leaders are referred to with numbers 1–21, their background information is provided in entirety, and the fields or units they led are not described.

## RESULTS

### Participant Characteristics

A total of 21 nurse leaders participated in the interviews. Considering their educational background, most were registered nurses (*n* = 17); the remainder were a paramedic, a public health nurse, a physiotherapist, and a midwife. Most participants were female (*n* = 17), representing midlevel or senior nursing leadership (*n* = 12) and frontline nursing leadership (*n* = 9). Their ages ranged from 38 to 62 years, with a mean age of 52. Participants’ nursing leadership experience ranged from 5 to 24 years, with an average of 15 years. Two participants reported a communication training background.

### Overview

**FIGURE 2:**
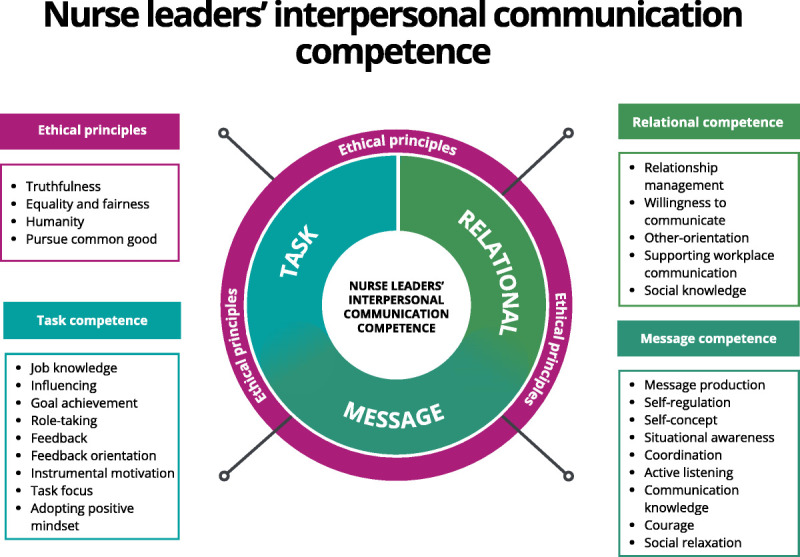
The outcome of the analysis. *Note*. The figure illustrates the outcome of the analysis, identifying four main categories and 27 subcategories. The analysis showed that nurse leaders’ interpersonal communication competence consisted of message competence, relational competence, task competence, and adherence to ethical principles. Each main category is represented alongside its corresponding subcategories.

The outcome of the analysis is illustrated in Figure 2. The results are presented in the following order: first, the ethical principles, followed by the nurse leaders’ interpersonal communication competence categories: message competence, relational competence, and task competence.

### Ethical Principles

Nurse leaders consider ethical competence at the core of interpersonal communication competence, establishing the key principles that guide competent communication. Ethical principles were categorized into four categories: truthfulness, equality and fairness, humanity, and the pursuit of the common good. As an ethical principle, truthfulness refers to honest and open messages and is fundamental to the functioning of relationships. It ensured the message was received as intended and did not cause unnecessary confusion or misunderstandings. For relationships, it was essential to create a psychologically safe communication climate. Equality and fairness were also key ethical values supporting this climate. Nurse leaders were expected to communicate in a way that showed equal consideration for all employees, fairness, and objectivity, as described by the following participant: “You must be able to remain neutral and fair, which means that in a large community like this, equality and fairness are particularly important.” (NL20)

Regarding humanity, participants emphasized the significance of being understanding and respectful, which affected building a collaborative communication climate and humane management. Respectfulness was described as an ethical action, considering the effect of communication on others and to avoid offense or harm. It had a positive effect on relationships and reduced conflicts. They also emphasized pursuing the common good, meaning a commitment to actions benefiting the organization, its members, patients, and the profession.

### Message Competence

The behavioral dimension of message competence includes creating and conveying messages, active listening, coordinating, and self-regulation in the sense of planning one’s own communication. Nurse leaders highlighted message production: the ability to create clear, structured core messages and convey messages understandably. They considered argumentation to be the single most important communication skill. It included the ability to concretize and illustrate messages as required by the context. This was closely linked to the ability to give meanings, structuring messages, and choosing words.

In a way, it really starts with the kind of written communication you use, the structure of your messages, how you present your point, and the words you choose… all of these have a huge impact on whether your message gets through or not. (NL04)

The above quotation also illustrates how nurse leaders need to be able to plan their own communication behavior and develop strategies to achieve their goals. Designing messages and choosing communication channels required anticipating the audience’s specific needs and reactions. Conveying messages also required the ability to identify relevant information for their own leadership area and their employees. From the perspective of the cognitive dimension, adjusting communication required nurse leaders to have strong situational awareness, which included understanding not only the communication environment but also the timing and the target audience’s needs. Nurse leaders described situational awareness using terms such as “intuition,” “sensitiveness,” or “antennae,” as described by the following participant: “You really must think every day about how you say things, what methods you use, and what forms of communication you choose to adopt. It is like being constantly on alert, with your antennae up all the time.” (NL17)

Nurse leaders also needed skills to coordinate their communication. They needed to initiate and structure conversations, select and frame topics, and summarize and close discussions. Coordination also included negotiating, building consensus, refocusing the discussion, handling questions, and managing turn-taking while ensuring all participants were engaged.

Alongside verbal messages, nurse leaders described the importance of nonverbal messages, such as facial expressions and gestures, as essential in fostering reciprocity and expressing their own thoughts and feelings. As a metacognitive skill, self-regulation was regarded as the ability to monitor and regulate their own verbal and nonverbal communication. Self-regulation involves a clear self-concept, including understanding oneself as a communicator, self-awareness, and the ability to recognize emotional states. It requires understanding one’s communication style, strengths, areas for development, and values.

Nurse leaders highlighted that good communication requires active listening and sensitivity. They described active listening as a comprehensive process that includes listening as well as receiving and interpreting messages. It involves expressing active participation and receptivity, presence, and accessibility. In addition, nurse leaders need to be able to monitor their partners, the situation, and the environment.

As a basis for message competence, the nurse leaders stated that creating and conveying messages and coordinating interaction required an understanding of communication: “First, you need theoretical knowledge of communication—what constitutes good communication and how it is structured—and then, you also need basic communication skills.” (NL05)

As a cognitive dimension, communication knowledge was seen as a fundamental element in enabling nurse leaders to effectively navigate complex organizational communication environments. Understanding communication also included understanding the different functions of communication: presenting and illustrating, arguing, meaning-making, active listening, and communication in different channels. Furthermore, participants highlighted the understanding of communication styles and strategies along with communication theories and terminology.

Regarding the affective dimension, courage to communicate and social relaxation were considered significant. Nurse leaders required the courage to communicate to express their thoughts—which was seen as a fundamental aspect of the role of a nurse leader—while social relaxation reflected their overall competence and the ability to adjust communication.

### Relational Competence

In the behavioral dimension, nurse leaders need skills in relationship management and supporting workplace communication. The most critical relational skill was relationship management: The nurse leader’s ability to establish and maintain relationships. The ability to foster trust and nurture mutual respect through communication and a sense of appreciation was vital in developing relationships. To manage relationships, participants described the nurse leader’s ability to express empathy as crucial alongside skills of supportive communication:

What is important in working life is having compassion, empathy. As a leader, this means understanding the emotions, emotional states, and events that may be connected to what has happened, even when discussing something that may not have gone as planned. (NL03)

Participants highlighted the need to build relationships with employees and stakeholders and to network. In the affective dimension, this required the willingness to communicate and understand one’s communication partner, considering different perspectives, regarded as other-orientation. These were essential for collaboration, maintaining good stakeholder relations, and gaining a comprehensive overview. At the behavioral level, nurse leaders in multilevel relationships need to manage tensions between distance and intimacy, privacy and openness, and formal and informal relationships. For example, managing tensions requires regulating how much personal information to share: in one situation, it might be necessary to maintain distance, while in another, sharing private information could act as an icebreaker.

Relational skills were needed to handle challenging communication situations and resolve conflicts. Effective conflict resolution resulted from the interplay between relationship management and other skills. Active listening was crucial to ensure all parties felt heard. Conflict resolution also requires coordinated and planned communication, along with social knowledge and situational awareness. As a cognitive process, mental preparation was seen as critical, but strong preconceptions could also hinder success. Adjusting communication was also highlighted. Changing strategies quickly when initial plans did not work. Collaboration skills also encompass diverse relational skills, including relationship management, supporting teams, shared decision-making, negotiation and conflict resolution, and networking.

Nurse leaders needed to lead and support staff and communities in interaction. They required a proactive approach to create opportunities for mutual interaction. Participants linked nurse leaders’ ability to support workplace communication to the engagement and empowerment of nursing staff. Building and maintaining a positive communication climate required specific skills and was fundamental for supporting workplace interactions. Nurse leaders must monitor the work community and appropriately address diverse group phenomena. In the case of problems within the community, they needed to guide the interaction.

From the perspective of the cognitive dimension, managing relationships and supporting workplace interaction requires social knowledge regarding human and group behavior. Participants described the need for a broad understanding of human behavior, different personalities, communication styles, and needs. Constantly changing work communities requires nurse leaders to understand and recognize group phenomena to support work–community interaction as described by the next nurse leader:

These interaction situations vary greatly. For example, when a new unit is established, when a unit has existed for a long time, when two units merge into one, or when a unit is shut down, the interaction within the work community changes accordingly. (NL07)

As described above, nurse leaders need to understand the relationships between people, including their nature, tensions, and characteristics. It also comprised an understanding of the sociocultural factors that influence communication. Furthermore, nurse leaders need to comprehend the organization’s communication, including its culture, norms, and values.

### Task Competence

In the cognitive dimension, nurse leaders considered in-depth knowledge of the working environment as the cornerstone of task-related communication competence. Job knowledge was imperative for planning and implementing communication in an organizational setting, creating a professional image, as well as acting as a nurse leader. It included understanding the expertise required by their leadership area and the operational dynamics of nursing. Understanding the structure and functioning of the organization, as well as the organization’s communication practices and channels, gave leaders insight into the organization’s information flow. Complex and multidisciplinary healthcare organizations also need knowledge of the work community and its constituents, along with an awareness of relevant stakeholders and networks.

Regarding the behavioral level, the skills required for task competence included influencing, striving goals, taking on roles, and giving and receiving feedback. Nursing leadership demands the ability to influence, motivate, and engage staff, partly achieved through the nurse leader’s communication. It was crucial for nurse leaders to set and achieve goals, including sharing their own or the organization’s visions. Participants considered the nurse leader’s ability to communicate the vision particularly significant. Consequently, the ability to communicate vision and influence was seen as partly a function of the nurse leader’s ability to give meaning.

Nurse leaders needed to take on roles to act in a variety of functions and fulfill the associated responsibilities, duties, and behaviors. Participants described not only the nurse leader’s role but also roles associated with the work, such as colleagues, employees, and profession representatives. Being in a leadership role guided how nurse leaders interacted and communicated, which may have differed from the leader’s own personality.

Many participants considered feedback skills as a vital task-related skill. Nurse leaders must recognize where feedback is needed and give it appropriately and at the right time. They saw that nurse leaders had to be able to actively provide positive feedback—especially to their direct employees—but also constructive feedback when necessary. Giving constructive feedback also requires message competence in tailoring the feedback individually and sticking to the point without getting personal. From the perspective of the affective dimension, good feedback skills require an orientation toward feedback. According to the nurse leaders, feedback orientation was central to developing interpersonal communication competence.

Furthermore, all nurse leaders described communication as the cornerstone of nursing leadership, and leadership occurs through communication. Communication had to be seen as an important tool for the leader and was, therefore, described as instrumental motivation. For example, attitudes toward communication were described as “What I try to think about is the absurd situation where a nurse leader lacks the motivation and attitude for communication. How can you be a nurse leader without that?” (NL18)

Nurse leaders must be task-focused, adopt a positive mindset, and communicate an optimistic outlook. The right mindset was essential for setting goals and influencing others, along with maintaining a forward-looking perspective. When nurse leaders perceived their motivation or attitudes needed improvement, they had to be able to modify their own attitudes and motivation. For example, in change management, participants described the need to develop self-motivation and align attitudes with organizational goals before communicating changes to staff.

## DISCUSSION

This study’s purpose was to describe nurse leaders’ perceptions of interpersonal communication competence. The results showed that interpersonal communication competence in the nursing leadership environment is a complex and adaptive phenomenon. This study aimed to summarize the phenomenon’s key elements and explore its description through the cognitive, behavioral, and affective dimensions identified in interpersonal communication competence theory (Spitzberg, 2022). Through our examination of this theory, we identified these dimensions across all categories of nurse leaders’ interpersonal communication competence. The findings are aligned with best practices and are intended to be instructive across various settings.

The research design followed a novel approach by analyzing the outcomes of the deductive phase through the lens of a communication theory. Based on our experience, this complex setting significantly enhanced our understanding of the phenomenon. It enabled us to adopt a multidisciplinary perspective and integrate diverse insights into our research. Problem-focused multidisciplinary research is considered imperative for generating comprehensive information on an identified knowledge gap (Carr et al., 2018; McKenna, 2020). The analysis was also facilitated by the rich research data collected. For instance, nurse leaders’ descriptions of tensions and tension management strategies are quite consistent with the tensions described in relational dialectics theory (Baxter & Montgomery, 1996; Johnson et al., 2018).

The study identified the ethical dimension as central to nurse leaders’ interpersonal communication competence. Although previously described (Spitzberg & Cupach, 2011), it has not been considered as a dimension in many studies. In this study, the nurse leaders described ethical principles as key principles of interpersonal communication, aligning with research on the importance of ethical competence in nursing practice and leadership. Understanding situations morally, using good moral judgment and intentions, and behaving ethically are seen as the basis of ethical action (Hansen et al., 2024; Zafarnia et al., 2017). This study suggests that these actions are close to the nurse leaders’ interpersonal communication competence, consistent with findings that effective leadership and communication promote nurses’ ethical competence (Poikkeus et al., 2020; Wiisak et al., 2024). Thus, nurse leaders’ interpersonal competence is vital for developing nurses’ ethical competence and ensuring high-quality and ethically sustainable nursing care.

Nurse leaders viewed communication as a crucial tool for nursing leadership. Their descriptions were closely related to relational leadership styles (Leclerc et al., 2022). They also saw interpersonal competence affecting staff motivation, engagement, and empowerment, consistent with previous research (Jankelová & Joniaková, 2021; Stillman et al., 2024). These influences are identified as the main outcomes of relational leadership styles (Hult et al., 2023), highlighting the connection between interpersonal competence and relational leadership.

An understanding of communication competence in nursing leadership is essential to retain competent nurses and ensure quality of care. There is also an acknowledged need to assess the current communication levels in nursing leadership (Fowler et al., 2021). Furthermore, it is important to study communication competence differences across sectors, which this study did not cover. Different nurse leader roles have also been identified as requiring different communication competencies and needing further research (Kämäräinen, Mikkola, Nurmeksela, Wright, & Kvist, (2024)).

### Strengths and Limitations

This study has both strengths and limitations. This study’s main strength was the rich data, providing deep insights into nurse leaders’ perceptions of interpersonal communication competence and achieving enabled saturation of key concepts. The multidisciplinary approach and research team strengthened the in-depth understanding of the topic.

Although interpersonal communication competence is a relational process and perceptions of competent communicators vary across contexts (Dannels, 2001; Horila, 2020), competence categories may be at least partially transferable to European organizational communication and leadership culture. However, the transferability is reduced by not reporting the participants’ leadership areas for confidentiality. Bias may occur when translating from Finnish to English, but this was minimized by regular discussions within the research team about translation.

## CONCLUSION

These results provide valuable insights into nurse leaders’ interpersonal competence, enhancing their role in nursing leadership. It provides an in-depth description of nurse leaders’ interpersonal communication competence and emphasizes the importance of the ethical dimension, supported by interpersonal communication competence theory. Due to the significant influence of nurse leaders’ interpersonal communication competence on staff, further research is needed to gain a deeper understanding of the phenomenon.

**Figure:**